# Validation of the Chinese version of the financial toxicity scale in patients with wet age-related macular degeneration

**DOI:** 10.1186/s41687-025-00909-x

**Published:** 2025-06-16

**Authors:** Xiuli Chen, Hong Bian, Zhifeng Wu

**Affiliations:** Jiangnan University, Wuxi No 2 People’s Hospital, No.68,Zhongshan Road, Wuxi, China

**Keywords:** Wet age-related macular degeneration, Financial toxicity, Reliability, Validity

## Abstract

**Objective:**

to confirm the validity of the Comprehensive Score for Financial Toxicity (COST) Scale in Chinese patients with wet age-related macular degeneration.

**Methods:**

A tertiary hospital in Wuxi’s ophthalmology outpatient clinic treated 217 patients with wet age-related macular degeneration (wAMD) were chosen using the convenience sample approach for a questionnaire survey between October 2023 and February 2024. The Chinese version of the COST and general patient information were included in the survey. Critical ratio analysis and correlation analysis were used to examine the items on the scale. The structural validity of the scale was evaluated using factor analysis, the reliability of the scale was evaluated using Cronbach′s α coefficient and retest reliability, and the content validity of the scale was evaluated using the Content Validity Index (CVI).

**Results:**

The item analysis results demonstrated that high and low subgroups could be identified using The COST scale’s Chinese translation (*P* < 0.01).; A linear positive correlation was observed between the scores of each item and the scale’s overall scores (*r* values of 0.243 ~ 0.878, *P* < 0.01), and the scores of factor 1, factor 2, and factor 3 showed a linear positive correlation with the total scores of the scale (accordingly, *r* values were 0.974, 0.505, and 0.300; *P* < 0.01). and the scores of each item were linearly and positively correlated with the scores of the common factors to which they belonged (*r* values of 0.642 to 1.000, *P* < 0.01). Content validity showed that the I-CVI of each item was 0.857 ~ 1.00, and the S-CVI was 0.974. Three metrics in all, with a cumulative variance contribution rate of 67.739%, were obtained through exploratory factor analysis. Each item’s loading on the corresponding dimensions varied from 0.638 to 0.954. The Cronbach’s α coefficient for the entire scale was 0.876(95% *CI*: 0.85–0.899). The reliability of the retest was 0.970 (95% *CI*: 0.936–0.990).

**Conclusion:**

The Chinese version of the COST scale shows potential applicability pending further validation.

## Introduction

Financial toxicity is defined as the process of disease treatment that brings to patients.

objective material losses and subjective psychological distress, and has an impact on the patient’s physiological and psychological quality of life and clinical outcomes [1]. An aging population and advances in medical technology have extended life expectancy that could have been shortened, and the prevalence of chronic diseases is increasing. healthcare costs associated with health problems and systemic low wages put people with chronic diseases at higher risk of financial hardship. “Financial toxicity” might result from the opportunity costs and healthcare expenses related to chronic illnesses [2]. negatively affecting patients’ adherence to treatment [3], prognosis [4], and quality of life [5, 6], and creating barriers to successful chronic disease management.

One of the three primary eye disorders that the World Health Organization has prioritized for the prevention of blindness is age-related macular degeneration (AMD) [7], a chronic, progressive neurodegenerative disease that affects the macular portion of the retina [8, 9]. The late stage of this condition, called wet age-related macular degeneration (wAMD) or neovascular age-related macular degeneration (nAMD), is a major contributor to vision impairment or blindness in the elderly [10, 11]. In patients with wAMD, Intravitreal injection of anti-vascular endothelial growth factor (Anti-VEGF) can improve visual function and restore the macular region’s structural structure. It is a first-line therapy for wAMD patients [12, 13]. Anti-VEGF therapy, requiring repeated monthly treatments, has significantly improved patient outcomes [14]. However, frequent and prolonged Anti-VEGF treatments and their incurable nature lead to the accumulation of treatment costs indefinitely, similar to cancers that require long-term maintenance therapy, creating a unique pattern of “chronic high expenditures”. This creates a heavy financial burden for patients [15–18] reducing patient compliance and presenting challenges to wAMD treatment [19, 20]. The national impact of rising costs associated with wAMD management has been actively studied and has been the subject of policy interventions [21], but there is a lack of comprehensive assessment of the financial impact of cost-sharing for people with wAMD and their families. There are no validated tools to measure the financial toxicity of wAMD, and there is little data on the subjective experience and financial toxicity of people with wAMD.

The University of Chicago developed the Comprehensive Score for Financial Toxicity (COST) scale in 2014, It was the first tool used to measure financial distress in cancer patients. Patient-reported outcome measures form the basis of the scale, which has been validated in cancer patients [22, 23]. In 2017, Chinese scholars translated the source scale back-to-back and then jointly negotiated to form the first draft of the Chinese version of the scale; English teachers with senior titles were invited to translate the first draft of the Chinese version; a person with visiting experience in an English-speaking country was invited to compare and debug the translated Chinese version, the back-translated English version and the original English version of the scale, so as to make the Chinese version of the scale as close as possible to the original English version of the scale in terms of content, semantics, and format. Six senior oncologists were invited to evaluate the translation of each item in the first draft of the Chinese version of the scale and whether the words and phrases were in line with the Chinese expression habits, and then revised to form the final Chinese version of the COST [24]. It shows good reliability and validity in cancer population. Studies have shown that the COST scale can also be applied to chronic disease populations beyond cancer patients [25], Patel [26] et al. evaluated the applicability of the COST scale in non-cancer chronic diseases such as diabetes, showing good cross-disease validity. It indicates that the scale has a well-developed value system, which can avoid the validity controversy caused by the weak theoretical foundation of the new instrument and enhance the comparability of cross-studies. Although the scale has strong scientific rigor and practical feasibility, it has not been validated in populations other than Chinese cancers. The purpose of this study is to confirm among wAMD patients the reliability and validity of the COST scale’s Chinese translation. Initially, one item’s wording was altered to reflect respondents’ “disease” instead of “cancer,” adapting the survey to non-cancer contexts. This study validated the Chinese version of the Financial Toxicity Scale in patients with wet age-related macular degeneration, aiming to provide a basis and assessment tool for studies related to financial toxicity in patients with wet age-related macular degeneration.

## Subjects and methods

### Design and participants

During October 2023 to February 2024, individuals with wAMD receiving anti-VEGF medication treatment at a tertiary hospital in Wuxi served as study subjects. Inclusion Criteria: (1). getting anti-VEGF medication treatment after being diagnosed with wAMD. (2). Willing to participate, with intact cognitive function and no communication barriers. Exclusion Criteria: (1). Receiving anti-VEGF drug treatment for the first time. (2). Language communication disorders, cognitive impairments, or a history of mental illness. (3). Severe vision impairment from eye diseases or retinal diseases other than AMD, such as diabetic retinopathy, causes macular edema. (4). refusal to take part in the study or removal from it while it is underway. Prior to the study’s start, the Jiangnan University Medical Center’s Ethical Review Center reviewed and approved it under review approval number (2023) Ethical Review No. (Y-102). The Declaration of Helsinki’s tenets were adhered to during the entire investigation.

### Calculating the sample size

There are 11 total items in the Chinese version of the COST scale, and it has been suggested that, when applying the scale, the sample size be approximated to be five to ten times the number of elements in the scale [27].and the sample size is enlarged by 20% to allow for participant shedding. A minimum of 66 people must attend. In this study, A subsample of 20 participants was considered to fulfill the conditions for assessing test-retest reliability [28], After 3 weeks, Twenty patients were chosen at random to retake the COST Scale in Chinese, and the two measurements were performed in the same environment.

### Research tools

(1) General Information Questionnaire: Created by the researchers, it includes information on the affected eye, age, gender, education level, occupation, marital status, length of the disease, frequency of injections, and the existence of additional chronic conditions.

(2) The Chinese Version of the COST Scale: The first instrument to measure financial distress in cancer patients was the COST scale, created by Souza^22^ et al. for patients with advanced cancer in the USA. There are 11 objects total, spread over three dimensions: financial outlay (one item), financial resources (two items), and psychological reaction (eight elements). Yu^24^ et al. translated the scale into Chinese and validated it in cancer populations. The findings indicate that the tumor population has good psychometric features (Cronbach’s α = 0.889). The Likert5 scale, which runs from 0 (“not at all”) to 4 (“very much”), is used for the same grading criteria as the original COST scale in Chinese. The scale has a total score of 0–44. Lower scores indicate higher financial toxicity. Patients rated their experiences over the past seven days.

### Survey methods and quality control

Before the investigation starts, the patient is informed of the goal and importance of the study, which is carried out in the ophthalmology outpatient clinic, and the patient’s consent is obtained. Strictly adhering to inclusion and exclusion criteria, a paper-based questionnaire survey was conducted on-site. The distribution and collection of questionnaires were completed by the researchers and another trained team member. One-to-one completion ensured the accuracy and completeness of the data, which was collected on the spot after completion. Two individuals independently verified and entered the data to ensure accuracy. With a sample size ten times larger than the total number of items in the questionnaire, 220 questionnaires were distributed, and 217 were recovered. The effective return rate of the questionnaire in this study was 98.6%.

### Validation of reliability and validity

(1) Each item on the scale was tested for homogeneity and discrimination using item analysis. The results of the COST scale in Chinese were sorted from high to low using the crucial ratio approach. The top 27% of scores were designated as high groups, and the bottom 27% as low groups. The scores of the two groups were then compared. The relationship between the scores of each item and the total score was determined using Pearson correlation analysis; the greater the *r* value, the stronger the link. (2) The scale’s content validity was assessed using the content validity index’s content, and 7 experts (2 ophthalmology medical experts, 2 clinical pharmacy experts, and 3 ophthalmic nursing experts) were asked to establish a group of experts in order to assess the scale’s content validity, and at the scale level, the mean content index (S-CVI) and the content validity index (I-CVI) were computed. Exploratory factor analysis (EFA) was utilized to evaluate the scale’s structural validity, principal component analysis (PCA), and variance The number of factors and items deleted were determined using the maximum orthogonal rotation approach based on the following criteria.: ① The load of items on a common factor > 0. 400 is the factor attribution criterion; ② If there is a double load (at the same time on 2 or more common factors, the load value > 0. 400), considering the retention or deletion of entries in combination with expertise. (3)Cronbach’s α coefficient was used to assess the scale’s internal consistency, Cronbach’s α > 0.7 indicates good reliability [29]. We employed the intraclass correlation coefficient (*ICC*) to assess the reliability of the retest. after a 3-week gap, reinvestigated the individuals who had improved compliance using the same scale. Evaluation of retest reliability using *ICC* correlation coefficients, which was obtained from the two outcomes. Statistics were deemed significant if *P* < 0.05. An acceptable result is defined as an *ICC* > 0.7 [30].

### Statistical methods

Two researchers entered data into Epidata 3.1, and IBM SPSS Statistics 26.0 was used for analysis. Pearson correlation analysis was utilized for correlation analysis, and the t-test was employed to compare groups. Continuous data were presented as ($\bar{x}\pm\:s$). Consider the difference to be statistically significant if *P* < 0.05.

## Results

### General information of patients

Table 1 displays the study participants’ characteristics. The study surveyed 217 wAMD patients, 117 men (53.90%) and 100 women (46.10%), whose average age was (71.41 ± 8.71) years. Most patients were married (92.2%), retired (77.0%), had health insurance (94.0%), had monocular vision loss (80.2%), and over half of them began treatment in the first year (50.2%). Participants mostly live with their families (89.9%) and have other chronic conditions (68.7%).

**Table 1:** General data of patients with wAMD (*n* = 217)

Characteristics	*n*	( %)
**Age**	71.41 ± 8.71 [range 45–90]	
**Gender**		
male	117	53.90%
female	100	46.10%
**Marital Status**		
married	200	92.20%
Single (unmarried, divorced, widowed)	17	7.80%
**Education Level**		
Primary school or below	38	17.50%
Middle school or technical school	94	43.30%
High school	41	18.90%
University and above	44	20.30%
**Employment status**		
Retired	167	77.00%
Employed	12	5.50%
Unemployed	38	17.50%
**Household Monthly Income per Capita(CNY)**		
Below 1000	2	0.90%
1000–3000	44	20.30%
3000–5000	104	47.90%
Above5000	67	30.90%
**Insurance type**		
UEBMI	104	47.93%
URBMI	55	25.35%
NCMS	45	20.70%
Self-paying	13	6%
**Living Situation**		
Living alone	22	10.10%
Living with family	195	89.90%
**Affected Eye**		
Right eye	77	35.50%
Left eye	97	44.70%
Both eyes	43	19.80%
**Number of Injections**		
2–3 times	109	50.20%
4–6 times	51	23.50%
> 6 times	57	26.30%
**Presence of Other Chronic Diseases**		
Yes	149	68.70%
No	68	31.30%

Note: UEBMI = Urban Employees’ Basic Medical Insurance Scheme; URBMI = Urban Residents’ Basic Medical Insurance Scheme; NCMS = New Cooperative Medical Scheme

### Content validity

There are eleven items on the COST scale’s Chinese equivalent. One of the items in the survey was reworded to reflect the respondent’s “disease” rather than “tumor” before it was launched, making it non-tumor related. Seven specialists in clinical ophthalmology, ophthalmic nursing, and pharmacy departments were requested to assess the scale’s content relevancy. The matching values 1, 2, 3, and 4 were assigned the following grades: “not relevant,” “somewhat relevant,” “relevant,” and “most relevant” using the Likert4 grade scoring technique. Experts are asked to rank each item’s content relevance. Determine how many experts have a rating of 3 or 4. The scale-level content validity index (S-CVI) and the content validity index (I-CVI) for every item were computed. The Chinese version of the COST scale has an I-CVI that varies from 0.857 to 1.00, with an S-CVI of 0.974.

### Item analysis

(1). Discrimination: The Chinese COST scale’s scores were ranked from high to low, with the high subgroup being the first 27% of scores (> 27 points) and the low subgroup being the last 27% of scores (< 19 points). Then, two independent samples t-tests were performed on these two groups, and *t* The findings indicated that each item’s scores in the low and high subgroups differed statistically significantly when compared with the total scores of the scale (*P* < 0.01) (see Table 2). (2) Homogeneity test: the patients’ total COST score was (23.44 ± 6.70), and there was a linear positive correlation between the total scores of factors 1, 2, and 3 and the scale’s overall score (*r* values were 0.974, 0.505, 0.300, *P* < 0.01). The total scores of factors 1, 2, and 3 were, respectively, (15.22 ± 5.95), (6.30 ± 1.40), and (1.91 ± 0.64). Each item’s score showed a linear and positive correlation with the scale’s overall score (*r* values ranging from 0.243 to 0.878, *P* < 0.01). The scores of the 8 items of Factor 1 were linearly and positively correlated with the scores of this common Factor (*r* value of 0.638 ~ 0.888, *P* < 0.01), and the scores of the 2 items of Factor 2 were linearly and positively correlated with the scores of this common Factor (*r* value of 0.744 ~ 0.859, *P* < 0.01). The 1 item score for factor 3 was linearly and positively correlated with that public factor score (*r* value of 1.00, *P* < 0.01). (see Table 3).

**Table 2:** Comparison of scores of items of the Chinese version of COST between high - score group and low - score group ($\bar{x}\pm\:s$)

Item	high - score group(*n* = 59)	low - score group(*n* = 59)	t	*P*
item1	2.19 ± 0.66	0.63 ± 0.64	13.065	<0.01
item2	3.32 ± 0.84	0.86 ± 0.71	17.208	<0.01
item3	3.19 ± 0.84	1.15 ± 0.61	15.041	<0.01
item4	2.12 ± 0.91	1.73 ± 0.55	2.811	<0.01
item5	3.19 ± 0.92	1.92 ± 0.73	8.339	<0.01
item6	2.41 ± 0.77	0.27 ± 0.52	17.685	<0.01
item7	2.42 ± 0.67	1.69 ± 0.50	6.665	<0.01
item8	3.61 ± 0.53	1.20 ± 0.58	23.599	<0.01
item9	3.97 ± 0.26	3.51 ± 0.99	3.437	<0.01
item10	3.34 ± 0.76	1.34 ± 0.71	14.806	<0.01
item11	2.47 ± 0.65	1.36 ± 1.74	8.726	<0.01

*Note:P* < 0. 05 Indicates good discrimination

**Table 3:** Item - total correlations and item - to - own – factor correlations for the Chinese version of COST

Item	Average score (‾x ± s)	Scale total score (*r*)	factor1 (*r*)	factor2 (*r*)	factor3 (*r*)
item 1	1.47 ± 0.86	0.731^a^	0.749^a^	-	-
item2	1.99 ± 1.20	0.783^a^	0.835^a^	-	-
item 3	2.00 ± 1.03	0.822^a^	0.844^a^	-	-
item 6	1.24 ± 1.03	0.833^a^	0.852^a^	-	-
item7	2.02 ± 0.54	0.617^a^	0.638^a^	-	-
item 8	2.31 ± 1.07	0.878^a^	0.888^a^	-	-
item 10	2.24 ± 1.00	0.818^a^	0.822^a^	-	-
item 11	1.94 ± 0.71	0.636^a^	0.642^a^	-	-
item 5	2.55 ± 0.98	0.535^a^	-	0.859^a^	-
item 9	3.75 ± 0.75	0.243^a^	-	0.744^a^	-
item4	1.91 ± 0.64	0.300^a^	-		1.00^a^

*Note:*^a^*P* < 0. 01, - Indicates that no statistical analysis was performed

### Structural validity

**Fig. 1:**
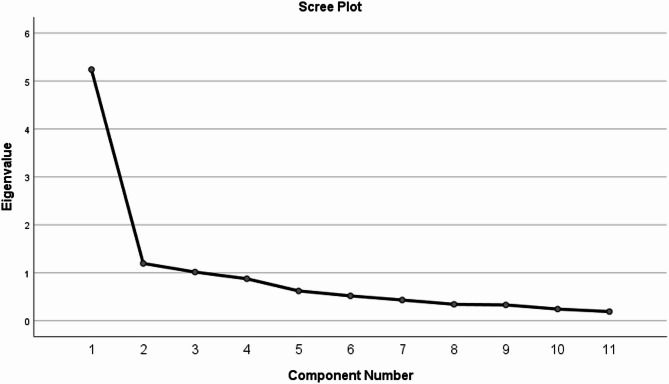
The Chinese version of the COST scale factor analysis screet plot

The scale met statistical prerequisites for factor analysis. according to the results of exploratory factor analysis (EFA), It showed that the variables had a substantial correlation, with a Bartlett spherical test value of χ^2^ = 1168.36 (*P* < 0.05) and KMO = 0.90. Three common components were extracted using principal component analysis (PCA), with a cumulative variance contribution rate of 67.739%. The eigenvalue > 1 was utilized as the criterion for factor inclusion. Eight items make up factor 1, two items make up factor 2, and one item makes up factor 3. Each item’s factor load in its common factor is between 0.638 and 0.954. The gravel diagram (Fig. 1) demonstrates that the fourth eigenvalue tends to be flat, suggesting that the three components are more appropriate. The specifics are displayed within Table 4.

**Table 4:** Reliability and rotated component matrixa of the Chinese version of the COST scale (*n* = 217)

Item	^b^Factor loading			Cronbach’s Alpha if item deleted
	Factor1	Factor2	Factor3	
item 8: I feel financially stressed	0.861			0.846
item 2: My out-of-pocket expenses are more than I thought they’d be	0.845			0.858
item 6: I am satisfied with my current financial situation	0.836			0.851
item 3: I worry about the financial problems I will have in the future as a result of my illness or treatment.	0.831			0.852
item 10: My disease or treatment has reduced my satisfaction with my present financial situation	0.786			0.852
item 1: I know that I have enough money in savings, retirement, or assets to cover the costs of my treatment	0.744			0.860
item 7: I am able to meet my monthly expenses	0.667			0.870
item 11: I feel in control of my financial situation	0.638			0.867
item 9: I am concerned about keeping my job and income, including work at home		0.878		0.890
item 5: I am frustrated that I cannot work or contribute as much as I usually do		0.683		0.877
item 4: I feel I have no choice about the amount of money I spend on care			0.954	0.884
**Variance explained %**	**67.739%**			
**Scale total mean score**	**23.44 ± 6.70**			
**Total Cronbach’s α of the scale**	**0.876**			

*Note:*^b^ Based on EFA

### Reliability analysis

(1) Cronbach’s Alpha: The overall Cronbach’s α coefficient for the Chinese version of the COST scale was 0.876(95% *CI*: 0.85–0.899). With correlations to the whole scale of 0.210 and 0.135, respectively, items 4 and 9 showed poor correlations. Both Cronbach’s α coefficients climbed to 0.890 and 0.884 after these items were removed. Table 4 provides further information.

(2) Test-Retest Reliability: 20 patients with good compliance were retested after three weeks. Considering the data analysis, Prior to and following the measurement, there was no statistically significant difference between the two groups (*t* = 1.406; *p* = 0.176). The two measurements have an *ICC* value of 0.970(95% *CI*: 0.936–0.990) and *P*<0.001. This suggests that the COST scale in Chinese has a fairly high test-retest reliability (see Table 5).

**Table 5:** Chinese version of COST retest reliability coefficients (*ICC*)

	First measurement	second measurement	ICC(95%CI)
item 1	1.50 ± 0.827	1.60 ± 0.754	0.921(0.812 ~ 0.968)
item 2	2.00 ± 1.170	2.00 ± 0.858	0.808(0.574 ~ 0.920)
item 3	2.20 ± 0.951	1.85 ± 0.933	0.815(0.446 ~ 0.932)
item 4	1.95 ± 0.759	2.00 ± 0.649	0.950(0.880 ~ 0.980)
item 5	2.50 ± 1.051	2.45 ± 0.945	0.874(0.710 ~ 0.948)
item 6	1.25 ± 0.910	1.20 ± 0.696	0.808(0.577 ~ 0.919)
item 7	1.90 ± 0.447	1.85 ± 0.366	0.850(0.666 ~ 0.938)
item 8	2.30 ± 1.031	2.20 ± 1.105	0.956(0.894 ~ 0.982)
item 9	3.70 ± 0.979	3.55 ± 0.826	0.848(0.661 ~ 0.937)
item 10	2.00 ± 0.725	1.90 ± 0.553	0.881(0.725 ~ 0.951)
item 11	2.00 ± 0.649	1.90 ± 0.553	0.863(0.690 ~ 0.943)
overall table	23.30 ± 5.814	22.50 ± 4.536	0.942(0.842 ~ 0.978)

*Note:ICC*>0.7 Indicates good consistency

## Discussion

Due to the special characteristics of wet macular degeneration, such as the need for on-time treatment on a monthly basis, the high price of therapeutic drugs, and the impossibility of curing the disease, Patients experience severe financial hardship as well as psychological distress. With the development of medical technology level, in the era of molecular targeting and immunotherapy, financial toxicity is a problem we cannot ignore [31]. At present, domestic financial toxicity-related research mainly focuses on the field of oncology, chronic disease financial toxicity is in its infancy [32], and there is no unified tool suitable for chronic disease financial toxicity measurement. The Chinese version of the COST Scale has been well applied to cancer patients, and this study used it on patients with wet macular degeneration, which provides preliminary evidence of psychometric suitability and offered an evaluation tool for the investigation of the financial toxicity of wet age-related patients in China. The tool will be used at a later stage to further explore relevant influencing factors and identify intervenable factors for relevant interventions to improve patient adherence and improve patient health outcomes. And in the future, other ophthalmic disease populations (e.g., glaucoma, diabetic retinopathy) can be added for relevant validation to further confirm the validity of the scale in different populations and provide a reliable basis for clinical practice.

In our study, we evaluated the psychometric qualities of the COST Scale in individuals with wAMD using the Chinese version. To the best of our knowledge, this study is the first to assess the reliability and validity of financial stress in connection to the treatment of wAMD. the mean participant financial toxicity score was 23.44 ± 6.70, which is slightly higher than the mean value of the source scale in the advanced cancer population. Consistent with the findings of Pavela [25] et al. that patients with wAMD and patients with other chronic conditions are experiencing financial toxicity, these patients experienced smaller financial toxicity effect sizes than cancer patients. Current financial toxicity assessments have not established universal clinical cut-off values, and financial toxicity thresholds need to be established in conjunction with a particular healthcare system’s payment policies and geographic economic level. There is a lack of cross-culturally validated criteria; secondly, different disease types may require differentiated grading criteria. In the future, data-driven methods can be used to establish clinically useful cut-off values. ROC analysis or latent class analysis can be performed on larger cohorts to establish clinically meaningful scoring thresholds, addressing the issue of missing thresholds in existing studies and enhancing the clinical translational value of research results.

By rewording one of the items to represent the respondent’s “disease” rather than “tumor,” the Chinese version of the COST Scale was altered to be unrelated to tumors. Each item’s I-CVI was graded by seven experts between 0.857 and 1.00. For the entire scale, the mean content index (S-CVI) was 0.974. Good content validity was observed for the scale [33].

The critical ratios for every item were statistically significant, according to the item analyses’ findings. Each item’s score and the scale’s overall score showed a linearly positive correlation, and there was a phenomenon of item4 being a common factor on its own. Item 4 and item 9 scores have a low r value in relation to the scale’s overall score, which should be considered to be deleted statistically. According to expert opinion, items 4 and 9 relate to patients’ concerns about medical expenses, and the family’s financial situation. Patients may fall into persistent anxiety over the cost of long-term treatment and reduced income, fearing that they will not be able to afford their medical expenses, and fearing that they will put heavy financial pressure on their families; this anxiety about financial hardship and concern about future uncertainty is a common and important psychological response. Despite the poor statistical indicators, they should be retained to ensure the theoretical integrity of the scale. Further analysis of the data showed that the *r* value of the scores of each item to the scores of its own common factor was high, which indicated that each item had a very good ability to differentiate between the psychological traits of the patients with wAMD and had a high degree of homogeneity with the overall scale, and therefore it was retained.

Factor analysis is a widely used and most reliable method to evaluate the structure of a scale. In this study, the structural validity of the scale was examined using exploratory factor analysis (EFA), and the results showed that there were three common factors with eigenroots > 1. 000, and each item had a high loading on the common factor to which it belonged, A more fine-grained reflection of the multidimensionality of medication pressures, indirect and psychological costs faced by wAMD patients. The validation of the Source Scale in patients with advanced cancer revealed a unifactorial structure, We think it may be related to the heterogeneity of the disease itself, sample characteristics, etc. Differences in symptom presentation and different pathomechanisms between wAMD patients and cancer patients may cause factor analysis to show different structures in different diseases. Cross-cultural revisions of the same scale, with different sources of funding, health insurance reimbursement rates, and policies for health care systems in different countries, may lead to different financial perceptions and different responses to finances among patients psychological distress which have significant differences in reimbursement rates. According to the 2022 report of the National Health Insurance Administration (NHIA) report, the average reimbursement rate for UEBMI expenses is 70–85%, while for URBMI/NCMS it is only 50–65% [34], which directly contributes to the rural patients’ increased out-of-pocket cost burden. Although critical illness insurance covers some high-value drugs (e.g., anti-VEGF drugs), the lag between catalog updates and geographic implementation differences (e.g., some provinces require patients to pay upfront) may still exacerbate financial toxicity. The Confucian tradition of family mutual aid ethic may also mask individual financial hardship, and family-based care models (e.g., family members accompanying patients to the doctor) may incur indirect expenditures such as lost wages and transportation costs in the absence of public care support. Uneven allocation of regional health resources forces patients to seek care across regions, indirectly pushing up non-medical expenditures, and barriers to cross-location settlement of health insurance exacerbate the economic risks of mobile populations.

Unlike the two-factor structure of the COST scale validated in chronic diseases such as diabetes, the three-factor model extracted in this study reflects more finely the multidimensional pressures of medication, indirect and psychological costs faced by patients with wAMD, and it is noteworthy that we found item 4 alone to be a common factor, which may be due to the fact that the medications used for the treatment of macular degeneration are expensive and need to be repeated several times month by month, and that there are limited therapeutic options available, and that the patient is cannot make other better choices, resulting in a higher cost burden for patients [17, 35].

The internal consistency and stability of the scale are reflected in scale reliability, internal consistency reflects the correlation of the scale items and is usually expressed by Cronbach’s α. Retesting reliability is typically used to represent stability, which is the consistency of the measurement results of the same study object on multiple occasions. and the reliability is considered to be good when Cronbach’s α is over 0.7. Cronbach’s α > 0.7 is considered to have good reliability. and In this study, The total Cronbach’s α coefficient of the scale in this study was 0.876, and the analysis of the reliability of each item found that the correlation coefficients of entries 4 and 9 with the total scale were lower, but did not affect the total Cronbach’s α coefficient of the scale. The Cronbach’s alpha coefficient of the total scale did not increase much after the deletion of entries 4 and 9, and after discussion among experts, item 4 and item 9 were retained. The reliability coefficients of the re-testing of the scale after a 3-week interval in patients with good compliance were 0.808 ~ 0.956, all of which were > 0.8, which indicated that the scale had good internal consistency and stability in general.

## Limitations

In our study, patient-reported financial data are susceptible to underreporting or social desirability bias, leading to data distortion. Patients may selectively conceal high levels of debt or exaggerate their economic capacity due to psychological factors such as “reluctance to disclose financial difficulties” or “desire to demonstrate strong coping abilities,” thereby affecting the reliability of scale scores and research conclusions. In the future, traditional face-to-face interviews or questionnaire completion in the presence of researchers could be replaced with anonymous self-completion methods. Alternatively, based on existing established scales, localized modified versions could be developed by integrating Chinese patients’ cultural background and economic environment. Emphasize data confidentiality in the scale instructions, use plain language to describe financial issues to reduce patient misunderstandings, and conduct pre-tests to verify the scale’s resistance to bias, ensuring its effectiveness in practical applications.

A limitation of this study was the exclusion of wAMD patients who were starting anti-VEGF therapy. This is mostly due to the fact that patients receiving treatment for the first time may not be completely informed of the available options and associated expenses. and the costs and psychological activities associated with the treatment are not fully reflected. All participants in this study were from a single medical center, limiting the generalizability of the findings to a broader group. In the future, the applicability of the scale in China’s diverse healthcare environment can be ensured through multi-center collaboration and stratified sampling strategies. Multi-center and community sampling can be used to construct a more representative cohort that covers the diversity of wAMD patients in China’s healthcare system. This will provide more reliable evidence to support the precise diagnosis and treatment of wAMD, the allocation of healthcare resources, and policy-making. Although this study initially supported the three-factor structure through exploratory factor analysis (EFA), sample dependence and risk of overfitting may affect the generalizability of the model. In future research, confirmatory factor analysis (CFA) should be conducted. Through independent sample CFA and fit index analysis (e.g., RMSEA, CFI, TLI), the reliability of the research conclusions can be ensured. In addition, the retest reliability assessment included only 20 participants with good adherence, which may overestimate the reliability indicators (e.g., *ICC* values), and compliance bias may make it difficult for the results to reflect response fluctuations in real clinical scenarios. Regarding validity validation, the COST scale was not tested for convergent validity, weakening the strength of evidence for its clinical relevance. Future correlations can be made with the Quality of Life Scale by calculating the Pearson correlation coefficient (*r*) between the total score of the target scale and the total score of the comparison instrument and testing for significance, which is used to assess convergent validity. Third, The current study employs a cross-sectional design, which can only capture the state of financial toxicity at a specific point in time and is unable to reflect its dynamic changes over time. However, patients’ financial toxicity is influenced by various factors, including treatment stage, disease progression, and adjustments to healthcare policies. In the future, repeated measurements using the Chinese version of the Financial Toxicity Scale could be conducted at key treatment milestones (e.g., at diagnosis, 3 months, 6 months, and 12 months into treatment) to observe trends in financial toxicity across different stages and analyze its association with treatment adjustments, disease remission, or progression. Finally, there are significant differences in insurance coverage, regional reimbursement rates, income levels, and urban/rural conditions, and these factors may lead to different financial toxicity faced by patients, which in turn may affect the effectiveness of the Chinese version of the Financial Toxicity Scale in measuring different subgroups. In the future, subgroups can be divided according to the type of insurance (e.g., commercial insurance, medical insurance, and no insurance), income level (high, medium, and low), and urban/rural status, and a multi-group CFA can be conducted to assess the changes in model fit by gradually restricting the equivalence of the model parameters across subgroups, and to determine whether the scale meets the measurement invariance requirements at different levels.

## Conclusion

The study’s findings demonstrate The Chinese version of the COST scale shows potential applicability pending further validation. It can be applied to assess the state of affairs and factors that influence the financial toxicity experienced by individuals suffering from wet age-related macular degeneration. The COST scale can be embedded in ophthalmology clinics, Collecting data on financial toxicity will help healthcare workers understand patients’ financial status and facilitate early identification and prevention of financial toxicity. Treatment items and cost expenditures may change over time. Continuous and dynamic assessment of the patient’s financial status is crucial, and medical professionals should continuously monitor the patient’s financial toxicity at various phases of treatment. Clinical doctors or financial advisors can classify the risk of financial toxicity into three levels—high, medium, and low—by combining the patient’s disease stage, treatment cost details, and scale scores. For high-risk patients, social work services should be initiated immediately, with professional social workers assisting patients in applying for charitable assistance funds, contacting community welfare agencies to obtain living subsidies, or connecting with public welfare organizations to resolve expenses such as nursing care and transportation. Financial advisors guide patients in applying for secondary reimbursement of medical expenses or major illness assistance subsidies based on their medical insurance type and regional policies. For example, they can assist rural patients in applying for New Rural Cooperative Medical Insurance for major illnesses or help low-income families apply for urban medical assistance funds. Clinical doctors collaborate with pharmacists, nutritionists, and others to make joint decisions with patients on adjusting treatment plans. While ensuring treatment efficacy, they prioritize medications covered by medical insurance (e.g., replacing high-cost imported drugs with domestically produced generic drugs of equivalent efficacy). For patients who have interrupted treatment due to financial pressure, they develop phased treatment plans to alleviate short-term financial burdens. Through longitudinal assessments, continuously monitor changes in patients’ financial toxicity, with scale retests conducted every 1–3 months. If the prognosis score remains unchanged, reassess the effectiveness of intervention measures and, if necessary, escalate intervention strategies (e.g., providing patients with information on long-term low-interest medical loans).

## Data Availability

All data generated or analyzed during this study are included in this published article.

## Abbreviations

AMD: Age related macular degeneration
wAMD: wet age related macular degeneration
Anti VEGF: Intravitreal injection of anti-vascular endothelial growth factor
COST: Comprehensive Score for Financial Toxicity
